# Interferon-stimulated genes and their antiviral activity against SARS-CoV-2

**DOI:** 10.1128/mbio.02100-24

**Published:** 2024-08-22

**Authors:** Ana Maria Ortega-Prieto, Jose M. Jimenez-Guardeño

**Affiliations:** 1 Departamento de Microbiología, Universidad de Málaga, Málaga, Spain; 2 Instituto de Investigación Biomédica de Málaga y Plataforma en Nanomedicina-IBIMA Plataforma BIONAND, Málaga, Spain; Albert Einstein College of Medicine, Bronx, New York, USA

**Keywords:** SARS-CoV-2, interferon, ISG, COVID-19, innate immunity

## Abstract

The coronavirus disease 2019 (COVID-19) pandemic remains an international health problem caused by the recent emergence of the severe acute respiratory syndrome coronavirus 2 (SARS-CoV-2). As of May 2024, SARS-CoV-2 has caused more than 775 million cases and over 7 million deaths globally. Despite current vaccination programs, infections are still rapidly increasing, mainly due to the appearance and spread of new variants, variations in immunization rates, and limitations of current vaccines in preventing transmission. This underscores the need for pan-variant antivirals and treatments. The interferon (IFN) system is a critical element of the innate immune response and serves as a frontline defense against viruses. It induces a generalized antiviral state by transiently upregulating hundreds of IFN-stimulated genes (ISGs). To gain a deeper comprehension of the innate immune response to SARS-CoV-2, its connection to COVID-19 pathogenesis, and the potential therapeutic implications, this review provides a detailed overview of fundamental aspects of the diverse ISGs identified for their antiviral properties against SARS-CoV-2. It emphasizes the importance of these proteins in controlling viral replication and spread. Furthermore, we explore methodological approaches for the identification of ISGs and conduct a comparative analysis with other viruses. Deciphering the roles of ISGs and their interactions with viral pathogens can help identify novel targets for antiviral therapies and enhance our preparedness to confront current and future viral threats.

## INTRODUCTION

Coronaviruses comprise a diverse family of positive-sense single-stranded RNA viruses responsible for a wide array of established and emerging diseases in both animal and human populations (1). Human coronaviruses have been historically linked to mild respiratory symptoms resembling the common cold, particularly in healthy individuals. However, they can also cause more severe clinical manifestations in vulnerable populations such as immunocompromised individuals, the elderly, and infants (human coronaviruses [hCoV]-229E, -NL63, -HKU1 and -OC43) (1). However, since different bat species serve as the primary natural hosts for most coronaviruses, they provide an ideal environment for recombination events among different viral species. These events have resulted in the emergence of novel coronaviruses capable of crossing species barriers and causing severe illness in both animals and humans (2).

Consequently, since the start of the 21st century, three previously unknown pathogenic coronaviruses breached the species barrier, leading to cases of severe illness in humans (3). The first, severe acute respiratory syndrome coronavirus 1 (SARS-CoV-1), was identified in 2002 in China, causing 8,000 confirmed infections worldwide with an estimated fatality rate of 10%. However, despite this epidemic being successfully controlled 1 year later and has not naturally re-emerged since, the widespread presence of SARS-like coronaviruses in bats circulating all over the world suggested the potential for another SARS outbreak (4). This prediction materialized in 2012 with the identification in Saudi Arabia of the Middle East respiratory syndrome coronavirus (MERS-CoV) in individuals with renal failure and severe pneumonia (5). Since then, MERS-CoV has been spreading and circulating, infecting at least 2,610 individuals and resulting in 858 fatalities (World Health Organization). In 2019, the SARS-CoV-2 was reported in China as the causative pathogen of the ongoing pandemic of coronavirus disease 2019 (COVID-19), rapidly disseminating to over 175 countries within 3 months (6, 7).

These emerging coronaviruses can induce severe pneumonia and acute respiratory distress, with SARS-CoV-2 posing particular concern due to its high contagiousness (8). To date, SARS-CoV-2 has led to more than 775 million cases and over 7 million deaths globally (World Health Organization), putting an unprecedented strain on healthcare facilities worldwide and causing an enormous impact on the global economy and society. Despite current vaccination programs (9), infections are still rapidly increasing (10), mainly due to the appearance and spread of new variants, different immunization rates, and the limitations of current vaccines to prevent transmission. This highlights the urgent need for pan-variant antivirals and treatments, especially amid the concurrent spread of additional viral pathogens causing respiratory diseases, such as the respiratory syncytial virus and influenza virus, further straining healthcare systems and resources (11). Moreover, the absence of effective antiviral compounds and treatments worsens the scenario, emphasizing the importance of continued research to identify and characterize more effective compounds and therapies against SARS-CoV-2 infection and COVID-19. Additionally, as described above, coronaviruses remain a persistent threat due to their ability to cross host species barriers and infect a broad range of organisms, heightening the risk of another SARS-like outbreak (4).

Viral infection initiates a continuous battle between the host’s defense mechanism, which aims to destroy infected cells or suppress viral replication, and the replicating virus. In this context, the interferon (IFN) response plays a decisive role during the innate immune response against pathogens. It operates following the sensing of pathogen-associated molecular patterns (PAMPs) by specific pattern recognition receptors (PRRs) encoded by the host, constituting the front line of defense against viruses (12). This coordinated response leads to the induction of an antiviral state involving the transcriptional upregulation of hundreds of IFN-stimulated genes (ISGs) with potential antiviral properties (13, 14). To date, dozens of human ISGs with antiviral activity have been identified, with much of their viral targets and functions yet to be fully understood (15, 16). Importantly, this protective mechanism can become a double-edged sword, as uncontrolled signaling can lead to dysregulated and prolonged inflammatory responses that may cause severe harm to the host, ultimately leading to severe disease and fatal outcomes (17).

To initiate successful infections, many viruses have evolved different procedures to evade this antiviral response by targeting specific elements of the IFN system (18). However, despite the presence of viral strategies to evade the host antiviral activities, the IFN system is still able to limit or prevent the viral infection of most viruses (19). Interestingly, despite SARS-CoV-2 developing mechanisms aimed at antagonizing the IFN response, involving viral components such as the non-structural proteins 1 (Nsp1) and 15 (Nsp15) and the open reading frames (ORF) 3b, 9b, and 6 (20), host cell recognition still leads to the secretion of IFN and subsequent expression of ISGs in human airway epithelial cells (21–26).

Interestingly, different studies have already demonstrated that Type I IFN treatment or pre-treatment has a protective effect in cells infected with SARS-CoV-2, indicating its potential to mitigate the severity of COVID-19 (27, 28). Furthermore, clinical evidence also supports the potential use of IFNs in treating COVID-19 patients, while a deficiency of type I IFN in the blood may be indicative of severe COVID-19 (29, 30). However, despite the potential importance of IFN treatment in the initial days of infection, its administration during the inflammatory and severe phases of SARS-CoV-2 infection could potentially exacerbate the condition, potentially leading to uncontrolled inflammatory responses and harmful effects (31–33). Recent research has also revealed significant deregulation of the innate immune response in severe cases of coronavirus infection (34), along with a notable role of anti-type I IFN autoantibodies in exacerbating COVID-19 severity (35). Remarkably, high rates of respiratory failure and severe pneumonia were documented in elder people and individuals with previous mild comorbidities, including diabetes mellitus, overweight, and hypertension (36), factors leading to a well-documented deficiency in the induction of IFNs in response to pathogen infections (37–39).

Within the intricate landscape of the IFN response, the induction of ISGs plays a fundamental role in shaping the antiviral defense. Interestingly, recent studies have shown that, in comparison to ancestral isolates, emerging variants of concern have developed increased resistance to IFNs. This enhanced resistance may be attributed to a reduced antagonism of ISG actions, underscoring the significant role of IFNs in the evolutionary trajectory of SARS-CoV-2 (40, 41). Over the past 3 years, substantial efforts have been directed toward identifying ISGs with antiviral properties against SARS-CoV-2. In this context, numerous studies have investigated the repertoire of ISGs across various infection models utilizing advanced technologies, such as microarray and RNA sequencing (RNA-seq), to analyze gene expression. Understanding the repertoire of ISGs with the potential to counteract SARS-CoV-2 is pivotal for the advancement of more effective therapeutic approaches against COVID-19.

The objective of this review is to synthesize the current understanding of ISGs and their crucial role in countering SARS-CoV-2 infection, shedding light on potential targets for antiviral drug development and immunomodulatory therapies. Within this scope, we will explore methodological approaches employed for identifying ISGs, detail the described mechanisms driving their antiviral activity against SARS-CoV-2, and compare their effectiveness and specificity across different viruses. Through a comprehensive analysis of the complex interactions between ISGs and SARS-CoV-2, this review aims to offer insights that can enhance our ability to confront not only the ongoing COVID-19 pandemic but also potential future viral threats.

## METHODOLOGICAL APPROACHES FOR THE IDENTIFICATION OF ISGs

Methodological approaches for identifying ISGs with antiviral activity have evolved significantly over the years, reflecting remarkable advancements in molecular biology and bioinformatics. These approaches are essential for unraveling the complex mechanisms of antiviral defense and comprehending the roles of ISGs in these processes. As our knowledge of the complex interplay between viral pathogens and host immune responses continues to expand, refining and diversifying techniques for ISG identification and characterization are pivotal in driving scientific progress in virology and immunology. Here, we will discuss some key methodologies utilized for the identification and characterization of ISGs.

### Transcriptomic profiling

The first step in the quest to identify potential ISGs with antiviral activity usually involves pinpointing genes whose transcription is upregulated after IFN treatment. In this context, transcriptomic profiling approaches, including microarray analysis and RNA-seq, have emerged as transformative tools to unravel the intricate molecular landscape underlying host–virus interactions. These methods support the discovery of novel ISGs and provide insights into the dynamics of gene regulation and regulatory networks governing antiviral responses. Importantly, high-throughput methods enable more effective comparisons of gene expression patterns across different cell types, tissues, and experimental conditions, elucidating the context-specific roles of ISGs in antiviral defense.

Detailed investigations into the transcriptional response to SARS-CoV-2 across various cell types and infection models have shown the significant induction of a subset of ISGs following low levels of different types of IFNs (22). Accordingly, studies scrutinizing alterations in gene transcription induced by SARS-CoV-2 in human small intestinal organoids have unveiled a broad array of ISGs and cytokines associated with different types of IFNs (42). Notably, single-cell transcriptomic analyses of intestinal organoids infected with SARS-CoV-2 have suggested that variations in ISG production among different cell types may contribute to the permissiveness of distinct cell types to SARS-CoV-2 infection (43). Furthermore, comparisons of the host transcriptomes in response to different pathogenic human coronaviruses have revealed a notably stronger induction of ISG expression caused by SARS-CoV-2 in comparison to SARS-CoV-1 in Calu-3 cells (44). Although these experiments alone do not allow us to evaluate the antiviral activity of the identified ISGs, they serve as the foundation for further exploration. Collectively, these findings underscore the intricate interplay between ISGs and viral infections, offering an important understanding of the host defense mechanisms against SARS-CoV-2 infection.

### Proteomics approaches

Proteomics techniques, including mass spectrometry-based proteomics and protein microarrays, offer a comprehensive view of protein expression and interactions. By analyzing the proteome of cells treated with IFNs, researchers can pinpoint proteins induced or modified in response to IFN stimulation, complementing transcriptomic analyses and unveiling post-transcriptional regulation of ISGs.

The analysis of confirmed COVID-19 cases using multiplexed high-resolution mass spectrometry-based proteomics revealed a vigorous antiviral response mediated by IFNs in the nasopharynx of individuals infected with SARS-CoV-2 (45). Interestingly, this approach identified numerous upregulated proteins previously not linked to antiviral responses. Notably, most of the proteins exhibiting significant increases in infected individuals were described in the Interferome database (46) as proteins whose expression was found to be induced after IFN treatment. These proteins include those synthesized by classical ISGs like the interferon-induced proteins with tetratricopeptide repeats (IFITs) proteins IFIT1, IFIT2, and IFIT3; the interferon-induced GTP-binding proteins MX1 and MX2; the interferon-stimulated genes 15 (ISG15) and 20 (ISG20) proteins; the probable E3 ubiquitin-protein ligases HERC5 and HERC6 proteins; and the 2′,5′-oligoadenylate synthetases 1 (OAS1) and 2 (OAS1) proteins (45). Trugilho et al. employed a label-free shotgun proteomics method to investigate the platelet proteome in individuals infected with SARS-CoV-2, uncovering a comparable subset of ISGs upregulated by SARS-CoV-2, including IFIT1 and IFIT3, ISG15, interferon‐induced transmembrane proteins (IFITMs), and the interferon-induced 35-kDa protein (IFI35) (47). Additionally, a quantitative proteomics-based approach using tandem mass tag (TMT)-labeling strategy of human hepatoma cells infected or not with SARS-CoV-2 demonstrated increased protein levels of various ISGs, including ISG15, at 48 h post-infection (48).

Similar to transcriptomic profiling approaches, proteomics techniques offer a deeper insight into host–virus interactions. Moreover, proteomics provides a unique opportunity to study post-transcriptional modifications, protein–protein interactions, and protein turnover rates, all of which contribute to the intricate regulatory mechanisms governing ISG expression and antiviral responses (49). Although exploration in these areas is still relatively limited, proteomics holds promise in unravelling the complex regulatory landscape of ISG expression, offering insights into how ISG activity is fine-tuned in response to viral infections and paving the way for innovative therapeutic approaches.

### Loss-of-function studies

Loss-of-function studies, such as clustered regularly interspaced short palindromic repeats (CRISPR)-based knockout or RNA interference approaches, play a pivotal role in systematically unraveling gene function on a genome-wide scale. By selectively inhibiting the expression of individual ISGs in cells treated or not with IFNs, researchers can identify ISGs with antiviral properties, shedding light on novel ISGs and their roles in antiviral defense pathways.

In a recent functional CRISPR/Cas9 screen targeting 1,905 ISGs in human epithelial lung cells to assess their impact on SARS-CoV-2 infection, researchers characterized the death domain-associated protein 6 (DAXX) as a potent inhibitor of SARS-CoV-2 (50). Additionally, Nchioua et al. showed that siRNA knockdown of the zinc finger antiviral protein (ZAP) significantly enhanced SARS-CoV-2 infection, particularly during IFN-γ treatment (51). Expanding on these findings, genome-wide CRISPR activation and knockout screens conducted in human lung epithelial cells revealed a spectrum of proviral and antiviral factors spanning interconnected host pathways. Notably, this approach led to the discovery of mucins, a group of glycoproteins, as a significant antiviral network that interferes with SARS-CoV-2 infection in different models of infection (52).

Building upon these methodologies, ongoing advancements in loss-of-function studies have the potential to enhance our comprehension of host–virus interactions and unveil pivotal regulators of viral replication.

### Gain-of-function studies

Gain-of-function studies involve the manipulation of candidate ISGs through overexpression or ectopic expression to assess their antiviral efficacy. These studies serve as complementary approaches to loss-of-function studies, providing direct evidence of the ISG-mediated antiviral effect. Moreover, gain-of-function studies aid in identifying ISGs with potent antiviral activity, offering promising candidates for therapeutic intervention. While individual ISG studies have proven effective in identifying antiviral properties, the scalability and throughput of this approach are limited. To overcome these constraints, researchers have developed high-throughput gain-of-function screening platforms, enabling the systematic evaluation of numerous ISGs simultaneously.

To elucidate the host antiviral response following SARS-CoV-2 infection, Martin-Sancho et al. conducted a comprehensive overexpression screen to evaluate the antiviral activity of 399 human ISGs. Their study revealed that viral restriction primarily occurred through endoplasmic reticulum- and Golgi-resident proteins, including bone marrow stromal antigen 2 (BST2/tetherin), which inhibited SARS-CoV-2 particle release but was counteracted by the viral Orf7a protein (53). Functional analyses also highlighted the potent antiviral activity of lymphocyte antigen 6 complex, locus E (LY6E), which efficiently inhibited viral entry mediated by the spike proteins of multiple coronaviruses infecting humans, including MERS-CoV, SARS-CoV-1, and SARS-CoV-2 (54, 55). Zang et al. performed an ISG screen leading to the identification of the cholesterol 25-hydroxylase (CH25H) as a potent antiviral protein against SARS-CoV-2 (56), while Khan et al. identified the IFN-inducible short isoform of human nuclear receptor coactivator 7 (NCOA7) as an ISG with antiviral activity against SARS-CoV-2 (57). Other studies described the antiviral effects of overexpressing IFITM proteins against SARS-CoV-2 (58, 59). The 2′-5′-oligoadenylate synthetase 1 (OAS1) was also described to restrict SARS-CoV-2 in an ISG overexpression screen using a library of >500 human ISGs on human lung cells (60). A later study using an adapted CRISPR-activation system also identified OAS1 as a protein with antiviral properties against SARS-CoV-2 (61). Moreover, a transposon-mediated gene-activation screen identified the antiviral activity of the p41 isoform of the invariant chain CD74 against SARS-CoV-2 infection (62).

Overall, gain-of-function studies offer a fundamental understanding of the antiviral potential of ISGs and their role in fighting viral infections, including SARS-CoV-2. These studies complement loss-of-function approaches by providing direct evidence of ISG-mediated antiviral effects. Through comprehensive overexpression screens and functional analyses, researchers have uncovered a multitude of ISGs with antiviral properties against the highly contagious SARS-CoV-2. These findings underscore the diverse arsenal of host antiviral mechanisms and underscore the potential of ISGs as targets for therapeutic interventions against emerging viral pathogens.

**Fig 1:**
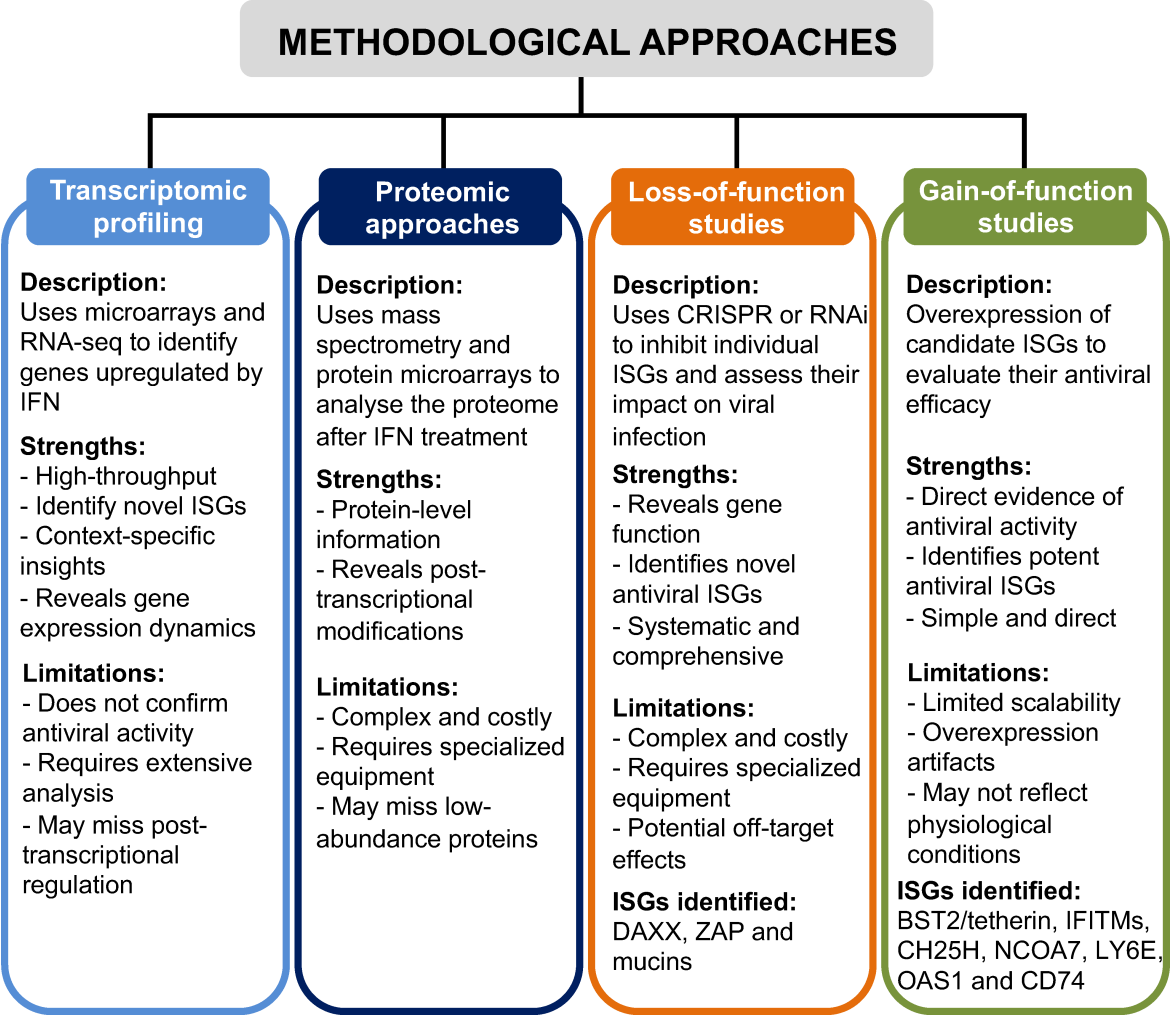
Comparative analysis of methodological approaches for identifying ISGs with antiviral activity, highlighting the key strengths and limitations of each method and the ISGs identified with antiviral activity against SARS-CoV-2.

In all these approaches utilized for the identification of ISGs and their antiviral activities (Fig. 1), the significant role of computational methods cannot be overstated. In this context, bioinformatics tools and databases are indispensable for analyzing gene expression data, predicting protein–protein interactions, and functionally annotating ISGs based on shared biological pathways or protein domains. Furthermore, the integration of multi-omics data through computational approaches enriches our understanding of ISG regulation and function, emphasizing the vital contribution of bioinformatics to advancing research in virology and immunology.

## ISGs WITH ANTIVIRAL ACTIVITY AGAINST SARS-CoV-2 AND COMPARATIVE ANALYSIS WITH OTHER VIRUSES

### Lymphocyte antigen 6 complex, locus E (LY6E)

LY6E has been shown to inhibit the spike protein-mediated membrane fusion of human coronaviruses (54–56, 63). It is constitutively expressed across different tissues in humans, including the lung, spleen, liver, brain, ovary, and uterus (64, 65), LY6E expression can be further induced by type I IFN, and its primary function has been mainly associated with modulating T-cell activation, development, and proliferation (54, 65, 66). Notably, experiments using murine models of infection revealed that the conditional absence of LY6E expression led to clinical illness and increased viral load post-SARS-CoV-2 infection (63). Further research aimed at elucidating the precise molecular mechanism through which LY6E inhibits viral entry is crucial for advancing our comprehension of cellular antiviral defenses.

LY6E is a pan-coronavirus antiviral restriction factor capable of inhibiting coronavirus spike-mediated fusion in the respiratory tract and VSV replication (63, 67). However, by contrasting its inhibitory activities in some viral infections, LY6E has been linked to increased susceptibility to infection by several enveloped viruses, including dengue virus (DENV), HIV-1, VSV, influenza A virus (IAV), and yellow fever virus (YFV) (16, 68). The different outcomes of LY6E, reported by various research groups worldwide, suggest cell type-dependent and virus-specific mechanisms (65).

### Nuclear receptor coactivator 7 (NCOA7)

NCOA7 expression comprises two different isoforms: a long isoform not inducible by IFNs and a short IFN-inducible isoform containing a distinct amino-terminal section of 25 amino acids (69–71). Known to interact with the vacuolar-type ATPase (V-ATPase) and promote lysosomal protease activity and endo-lysosomal acidification (72), the short isoform of NCAO7 demonstrated potent antiviral effects against SARS-CoV-2 in gene knockout and ectopic expression experiments in lung epithelial cells (57). Interestingly, the overexpression of the transmembrane protease, serine 2 (TMPRSS2), which enables plasma membrane fusion during viral entry, mitigates the blocking effect mediated by NCOA7 on SARS-CoV-2 (57). These findings further support the existence of different pathways for SARS-CoV-2 entry and support the hypothesis that the integration of the polybasic furin cleavage site into the viral spike might have prompted the utilization of TMPRSS2 during viral entry to evade the inhibitory activities of NCOA7 (57).

NCOA7 has been documented as a potent inhibitor of viruses utilizing the endo-lysosomal pathway for cellular entry, including SARS-CoV-2 and other coronavirus, IAV, and hepatitis C virus (HCV). Briefly, the interaction involving the V-ATPase and NCOA7 intensifies the protease activity of lysosomes, the acidification of vesicles, and the degradation of endocytosed material, ultimately hindering the fusion abilities of viruses tailored for the endo-lysosomal pathway, thus impeding infection (57, 72).

### Death domain-associated protein 6 (DAXX)

A functional CRISPR/Cas9 screen targeting 1,905 ISGs in human epithelial lung cells identified DAXX as a potent antiviral factor against SARS-CoV-2 (50). Interestingly, DAXX exerts its antiviral activity against SARS-CoV-2 early in viral entry to impede viral replication. This ISG, a scaffold protein found in promyelocytic leukemia protein (PML) nuclear bodies, undergoes cytoplasmic relocalization upon viral infection of basal levels of DAXX expression were potent enough to partially suppress SARS-CoV-2 replication, with overexpression further inhibiting viral infection. Interestingly, the viral papain-like protease (PLpro) counteracts the antiviral effects of DAXX by promoting its degradation (50).

DAXX’s antiviral activity was primarily recognized for its effectiveness in restricting viral infection against DNA viruses that replicate within the nucleus, such as human papillomavirus (HPV) (73) and adenovirus 5 (AdV5) (74). However, recent investigations have shown that this ISG additionally restricts SARS-CoV-2 and HIV-1, two RNA viruses replicating in the cytoplasm (50, 75, 76). Understanding the molecular mechanisms by which DAXX inhibits RNA viruses is essential for elucidating its broader antiviral functions.

### Invariant chain CD74 (CD74)

The invariant chain (CD74) performs a pivotal role in antigen presentation by facilitating subcellular trafficking and assembly of the major histocompatibility complex (MHC) class II (77). Expression of the p41 isoform was found to restrict the endosomal entry mechanism utilized by various viruses, thereby presenting additional functions beyond its canonical role in antigen presentation (62). Interestingly, a transposon-mediated gene-activation screen identified the antiviral activity of CD74 against SARS-CoV-2 (62).

The CD74 p41 isoform exhibits potent antiviral activity by targeting a diverse array of cathepsin-dependent viruses as part of the cellular defense mechanism. This includes various filoviruses and coronaviruses, broadening its impact across viral families and highlighting its significance in innate immunity against viral infections (62).

### Cholesterol 25-hydroxylase (CH25H)

CH25H is the gene responsible for encoding cholesterol 25-hydroxylase, an enzyme that facilitates the transformation of cholesterol into 25-hydroxycholesterol (25HC) (78). Zang et al. performed an ISG screen to identify CH25H as an ISG with antiviral activity against SARS-CoV-2. Furthermore, they demonstrated that this ISG impedes SARS-CoV-2 entry into the target cells by inhibiting spike-mediated membrane fusion (56).

CH25H exhibits wide antiviral properties in different enveloped viruses, including SARS-CoV-2, porcine epidemic diarrhea virus, and porcine transmissible gastroenteritis virus (13, 56, 79, 80).

### Mucins

Mucins encompass a group of heavily glycosylated proteins with a high molecular weight found on all mucosal surfaces, constituting a significant portion of the epithelial glycocalyx and mucus (81). Recent studies, including genome-wide bidirectional CRISPR screens, have identified and characterized the antiviral role of membrane-anchored mucins in different models of SARS-CoV-2 infection (52, 82). These findings highlight the pivotal role of endogenously produced and upregulated levels of membrane-tethered mucins in limiting SARS-CoV-2 entry, particularly during the initial stage of cell binding (52).

The antiviral activity described for mucins against the highly contagious SARS-CoV-2 is not unique. Results indicate an important antiviral effect for mucins across multiple viruses causing respiratory diseases, including MERS-CoV, SARS-CoV-1, and influenza virus (52, 83).

### Interferon‐induced transmembrane proteins (IFITMs)

IFITMs comprise a group of small proteins found in both the plasma and endo-lysosomal membranes of host cells. These proteins serve as potent antiviral factors with broad efficacy, primarily impeding the entry of a diverse range of viruses (84). However, published findings are contradictory concerning the antiviral activity of IFITMs on viral entry (59). Recent extensive research on coronaviruses has uncovered a multifaceted mechanism influenced by various factors, including distinct members of the IFITM protein family, the specific cell type utilized, and the experimental design (84). In summary, while endogenous IFITMs generally serve as cofactors facilitating the entry of SARS-CoV-2 (85, 86), the overexpression of particular IFITMs has been described to restrict viral infection (56, 58, 59, 85, 87).

The antiviral activities of IFITM proteins are not restricted to SARS-CoV-2. This family of IFN-inducible transmembrane proteins comprises factors that impede the entry of a plethora of relevant pathogens, including tick-borne encephalitis virus, SARS-CoV-1, HIV-1, IAV, Ebola virus, West Nile virus (WNV), and dengue virus (88–96). Interestingly, despite IFITMs were initially believed to primarily function by antagonizing virus–cell membrane fusion, recent studies have uncovered novel post-entry viral restriction mechanisms (89, 90).

### Bone marrow stromal antigen 2 (BST2/tetherin)

BST2/tetherin is a type II transmembrane protein recently recognized for its strong antiviral activity against SARS-CoV-2 infection in a large-scale gain-of-function analysis (53). Different experiments using immortalized cell lines and primary cells have further demonstrated that BST2/tetherin directly facilitates the tethering of SARS-CoV-2 viral particles to the surfaces of target cells (97). Moreover, SARS-CoV-2 infection has been shown to downregulate BST2/tetherin, thereby enhancing viral infection and spread (97, 98). Experiments investigating the impact of individual viral proteins on the antiviral activity of BST2/tetherin showed that viral ORF3a alters BST2/tetherin localization, ultimately leading to the subsequent enhancement in virus release. Additionally, it was described that viral spike is also involved in BST2/tetherin downregulation (99). Interestingly, a recent investigation showed that mutations incorporated within the spike protein found in the SARS-CoV-2 Omicron variant facilitate the viral evasion of this ISG (98).

BST2/tetherin was initially identified as a membrane protein whose expression is decreased by Kaposi sarcoma herpes virus, suggesting a potential antiviral role (100). Subsequent research has revealed that BST2/tetherin is a restriction factor that inhibits the release of a variety of enveloped viruses, including Ebola virus, SARS-CoV-2, hCoV-229E, SARS-CoV-1, and HIV. These viruses are known to bud either at the endoplasmic-reticulum–Golgi intermediate compartment or at the plasma membrane, where BST2/tetherin deploys its restrictive action by attaching their viral particles to either the cell membrane or intracellular membranes (53, 101–108). To counteract the antiviral properties of BST2/tetherin, most viruses have developed mechanisms to downregulate or alter its localization to prevent interference with virus budding. Primate lentiviruses, such as simian immunodeficiency viruses, typically employ Nef, a viral accessory protein, to direct BST2/tetherin for degradation through the lysosomal system (109, 110). However, HIV-1 and HIV-2 have developed different approaches due to a deletion in human BST2/tetherin that makes it resistant to Nef. While HIV-1 utilizes Vpu, HIV-2 employs the envelope glycoprotein to eliminate BST2/tetherin from the cellular sites of virus assembly (106, 109–112). Similarly, SARS-CoV-1 and SARS-CoV-2 use their spike glycoprotein and ORF7a to counteract BST2/tetherin (53, 98, 99, 113–115).

### Zinc finger antiviral protein (ZAP)

ZAP has been described to bind to the cytosine–phosphate–guanine (CpG) dinucleotides found in viral RNAs, consequently guiding them to degradation pathways with the assistance of cofactors like the tripartite motif-containing 25 protein (TRIM25) and the KH and NYN domain-containing protein (KHNYN) (51, 116–121). Additionally, it has been described that ZAP may be also implicated in the later stages of virus replication, although the precise mechanisms remain elusive (122, 123). Recent studies reported the expression of ZAP and its cofactors TRIM25 and KHNYN in human lung cells following SARS-CoV-2 infection and the antiviral role of this protein when overexpressed (51). These studies support the key role of ZAP in restricting SARS-CoV-2 due to the high presence of CG dinucleotides in the 3′ end region of SARS-CoV-2 (51, 122, 123). Indeed, Zheng et al. showed that ZAP interacts with the N gene found in SARS-CoV-2, a gene characterized by containing a higher frequency of CpG compared to other regions of the SARS-CoV-2 genome (124). However, the specific mechanisms governing the inhibitory activities of ZAP against SARS-CoV-2 are still to be completely investigated.

The human ZAP protein, which primarily targets CpG dinucleotides in viral RNA sequences, demonstrates the ability to impair the infection of various negative- and positive-sense single-stranded RNA viruses (116, 125, 126). Acting as a post-transcriptional RNA restriction factor within target cells, ZAP effectively targets viruses like filoviruses, coronaviruses, retroviruses, and alphaviruses (51, 116, 127, 128). However, its antiviral impact on double-stranded RNA (dsRNA) viruses remains unclear. On the other hand, certain viruses, such as DENV, Zika virus (ZIKV), herpes simplex virus type 1 (HSV-1), and YFV are unaffected by the presence of ZAP protein, allowing them to replicate normally (116, 129, 130).

### 2′-5′-Oligoadenylate synthetase 1 (OAS1)

OAS1, an important element of the innate immune response, plays a significant function in inhibiting viral replication by degrading viral RNA in conjunction with the ribonuclease L (RNase L) (131, 132). Wickenhagen et al. conducted an arrayed ISG expression screening leading to the identification of OAS1 as a potent ISG against SARS-CoV-2. Additionally, they performed a global mapping of viral RNA sites within SARS-CoV-2 potentially bound by OAS1. This analysis revealed a striking specificity in OAS1 binding, particularly targeting two conserved stem loops within the 5′-untranslated region (UTR) of SARS-CoV-2, which were identified as the primary viral targets (60). Interestingly, a specific Neanderthal OAS1 isoform has been suggested to confer protection against COVID-19 severity and susceptibility, particularly among people of European ancestry (133).

OAS1 displays broad antiviral activity across different viral families, including VSV, herpes simplex virus type 1 (HSV-2), SARS-CoV-2, and encephalomyocarditis virus (EMCV), underscoring its potent role in the innate immune response (60, 134–137). Interestingly, over the past decades, different studies have linked specific genetic mutations and single-nucleotide polymorphisms (SNPs) within the OAS family with a range of viral infectious diseases, including those caused by SARS-CoV-2, hepatitis B virus (HBV), and HCV (133, 138–141).

**Fig 2:**
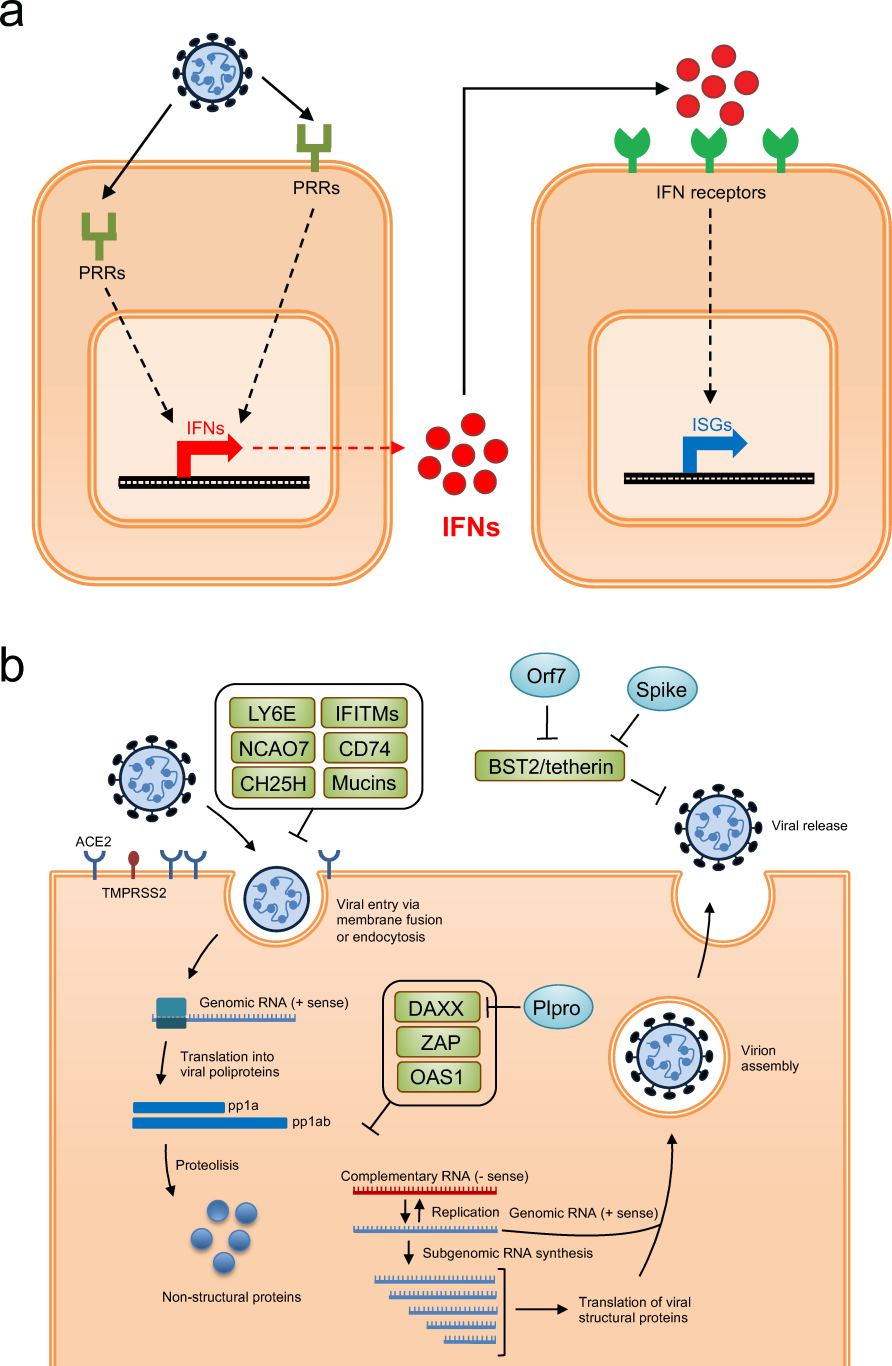
Schematic representation of the IFN response, the SARS-CoV-2 viral life cycle, and different ISGs identified with antiviral activity. (a) Schematic representation of the IFN response where the recognition of pathogen-associated molecular patterns (PAMPs) by specific pattern recognition receptors (PRRs) encoded by the host leads to the production and secretion of interferon, subsequently inducing an antiviral state involving the transcriptional upregulation of hundreds of IFN-stimulated genes (ISGs) with potential antiviral properties. (a) Schematic representation of the SARS-CoV-2 viral life cycle and various ISGs identified with antiviral activity. The ISGs identified are indicated in green boxes, and the viral proteins antagonizing their antiviral activity are indicated in light blue ovals.

The comparative analysis of ISGs and their inhibitory properties against SARS-CoV-2 (Fig. 2) in comparison to other viral pathogens reveals both commonalities and distinct features in host–virus interactions. To deepen our understanding of ISG-mediated viral susceptibility, it is essential to explore the specific factors that govern these interactions. For instance, while LY6E effectively inhibits coronavirus fusion and VSV replication, its varied effects on other viruses, such as DENV and IAV, highlight the influence of viral tropism and cellular context. Similarly, flaviviruses provide another illustrative example where ZAP can inhibit certain viruses within the family but not others (116, 142). While it has been described that some ZAP-resistant viruses encode proteins that actively antagonize the antiviral properties of ZAP, in other cases, the mechanisms by which these viruses evade ZAP inhibition remain unclear (116, 142–145).

Overall, these findings underscore the multifaceted nature of the host antiviral response, highlighting the intricate interactions between ISGs and SARS-CoV-2 and the importance of understanding them in combating viral infections. Furthermore, understanding the parallels and divergences between viruses sheds light on the broader landscape of host defense mechanisms and provides insights into the distinct challenges posed by different viral pathogens.

## CONCLUSIONS

Our understanding of the innate immune response against SARS-CoV-2 underscores the pivotal role of this system, particularly the IFN response, in controlling the virus and protecting against severe disease (146–150). However, despite recognizing the significance of the innate immune response, the specific mechanisms by which it controls the infection and the immunopathological processes induced by the virus remain incompletely understood.

In recent years, considerable attention has been devoted to identifying ISGs with antiviral properties against SARS-CoV-2. As a result, a restricted set of proteins has been acknowledged for their role in the overall antiviral effect, including the IFITM proteins, L6YE, ZAP, CD74, CH15H, mucins, DAXX, BST2/tetherin, OAS1, and NCAO7 (Table 1). Nevertheless, these identified factors only account for a fraction of the overall antiviral effect, indicating that there is still a rich and uncharted terrain of novel ISGs with antiviral potential against SARS-CoV-2. This knowledge gap may arise from limitations in screening methodologies, potentially overlooking ISGs weakly induced by IFN in specific cell types, despite their potential robust antiviral properties.

**TABLE 1:** List of ISGs identified with antiviral activity against SARS-CoV-2

Name	Abbreviation	Inhibited process	Antagonist	References
Bone marrow stromal antigen 2	BST2/tetherin	Viral release	SARS-CoV-2 spike and Orf7a	(97, 99)
Lymphocyte antigen 6 complex, locus E	LY6E	Viral entry		(63)
Nuclear receptor coactivator 7	NCOA7	Viral entry		(57)
Death domain-associated protein 6	DAXX	Early, post-entry step of the viral cycle	SARS-CoV-2 Plpro	(50)
Interferon‐induced transmembrane proteins	IFITMs	Viral entry		(59, 84)
Zinc finger antiviral protein	ZAP	Viral replication		(51, 122, 123)
2′-5′-Oligoadenylate synthetase 1	OAS1	Viral replication		(60)
Invariant chain CD74	CD74	Viral entry		(56, 62)
Cholesterol 25-hydroxylase	CH25H	Viral entry		(56)
Mucins	Mucins	Viral entry		(52, 82)

Many ISGs exhibit broad-spectrum antiviral activity, highlighting their versatility in combating infections across diverse viral families. For instance, IFITM proteins, renowned for inhibiting HIV-1 entry, also play a role in restricting the infection of diverse viruses, including IAV, Ebola virus, SARS-CoV-2, and dengue virus. Similarly, OAS1, a potent inhibitor of SARS-CoV-2, demonstrates antiviral effects against other RNA viruses such as VSV, HSV-2, and EMCV (60, 134–137).

However, despite their shared antiviral activities, certain ISGs may exhibit virus-specific or context-dependent effects, reflecting the complex interplay between host factors and viral pathogens. For example, while IFITM proteins demonstrate broad-spectrum antiviral activity, their effectiveness may vary among different viruses, suggesting tailored mechanisms of action against specific viral threats (88–95). Additionally, some ISGs may selectively target particular stages of the viral life cycle, resulting in varying effects on viral replication depending on the virus in question. Moreover, the comparative analysis between viruses highlights the adaptability of viruses in evading host immune responses and overcoming intrinsic antiviral defenses. Viral evasion strategies, such as the modulation of host factors or the acquisition of mutations conferring resistance to ISG-mediated restriction, underscore the ongoing battle between host immunity and viral pathogens. Understanding these evasion mechanisms is crucial for anticipating viral strategies and devising effective countermeasures to combat viral infections. Furthermore, since ISGs are pivotal in the initial antiviral defense by inducing an IFN-mediated antiviral state, they may contribute to sustained protection against reinfection (12–14). Elucidating the potential role of these ISGs in long-term immunity against SARS-CoV-2 is crucial for understanding the durability of immune responses following infection or vaccination. This aspect is particularly relevant given the emergence of viral variants and the evolving landscape of the COVID-19 pandemic (151).

Recent studies have highlighted the importance of genetic variations in ISGs, which can significantly impact the efficacy of the innate immune response against SARS-CoV-2. Polymorphisms in genes such as OAS1 have garnered particular attention due to their potential influence on COVID-19 outcomes. For instance, a specific Neanderthal-derived isoform of OAS1 has been associated with reduced severity of the disease in individuals of European ancestry (133). Similarly, polymorphisms in other ISGs may also play critical roles in determining the susceptibility and response to SARS-CoV-2 infection. These genetic variations can lead to differences in the expression levels, functionality, or inducibility of the ISGs, potentially altering the overall antiviral response. Identifying and understanding these polymorphisms can provide valuable insights into the mechanisms of host–pathogen interactions and inform the development of targeted therapeutic strategies.

The functional manipulation of these ISGs holds potential significance in therapeutic development against SARS-CoV-2 and other viral infections. Leveraging this knowledge, researchers may explore strategies to enhance ISG activities or bypass viral evasion mechanisms. For instance, small-molecule agonists targeting specific ISGs could potentially boost their antiviral efficacy. Alternatively, gene therapy approaches aimed at increasing ISG expression in vulnerable populations could bolster innate immune responses against viral pathogens.

In conclusion, while SARS-CoV-2 exhibited sensitivity to the antiviral properties of a specific subset of ISGs, it is important to recognize that this may represent just a fraction of the potential arsenal within the host’s innate immune system. The ongoing pursuit to identify additional ISGs and decipher their roles in combating viral infections underscores the depth and complexity of host–pathogen interactions. Moreover, by uncovering commonalities and differences across viral infections, we gain invaluable insights into the fundamental principles governing host–virus interactions. This comprehensive review provides a valuable understanding of the antiviral activities of ISGs and their role in fortifying host defenses against viral invasions. Identifying and elucidating these antiviral mechanisms provide a strong foundation for improving our preparedness to confront not just SARS-CoV-2 but also emerging viral threats with pandemic potential.
